# The Modern Movement in the Matter of the Standardisation of Disinfectants

**Published:** 1905-06-10

**Authors:** David Sommerville

**Affiliations:** Lecturer in Public Health, King's College, London


### Hospital Clinics.

### THE MODERN MOVEMENT IN THE MATTER OF THE STANDARDISATION OF DISINFECTANTS.

By David Sommerville, B.A., M.D., D.P.H., M.R.C.P., Lecturer in Public Health, King's College, London.

Since the early sixties when Pasteur, through his brilliant experiments, finally settled the ancient and vexed question of spontaneous generation, and opened the way for the various forms of research which are now being made in the field of biochemistry, great strides have been made in our knowledge of the intimate changes occurring in cell life. We have acquired new conceptions of fermentation, and those particular forms of it which obtain in the various types of infectious disease. The rise and development of bacteriology have revolutionised whole departments of biology and organic chemistry. The similarity in origin and action of digestive enzymes, and pathogenic toxins, have so closely brought together the previously separate fields of physiological chemistry and bacteriology that they now stand as inseparable correlatives of each other. When the history of this bio-chemical development is written the names of hundreds of eminent biologists and chemists, many of whom are still at work in various parts of the world, will follow those of Pasteur, Virchow, Koch, and Emil Fischer, on whose prophetic vision there early dawned the light, that behind the veiled processes of biology, were to be seen the old and familiar operations of chemistry and physics. With the discovery by Obermeir in 1873 of the spirillum of relapsing fever, of the tubercle bacillus by Koch in 1882, of the diphtheria bacillus by Klebs and Löffler, and of the tetanus bacillus by Nicolaier in 1884, and of the bacillus of Oriental plague by Kitasato in 1894, together with investigations of the chemical secretions of these organisms by a large number of workers on the Continent, in England, and in America, substantial material was obtained on which to construct a rational hypothesis for the operations of infection, and by which the previous shadowy theories of so-called miasmata were swept away. This new light disclosed numerous secrets—secrets of action and reaction between infective microbes on the one hand and blood and tissue cells on the other. It is now firmly established that certain diseases are the direct result of the action of the toxins of particular bacteria on the ultimate elements of our bodies—the tissue cells. An obvious corollary to this proposition is that in destroying the particular bacterium we destroy the corresponding toxins, and consequently prevent the specific disease. With hazy ideas in the past as to the nature of infection, there existed equally hazy ideas regarding the nature and mode of production of disinfection; and until recently no very marked distinction was made between a disinfectant (a body capable of destroying pathogenic germs), an antiseptic (a body capable of inhibiting the growth of micro-organisms but not of completely destroying them), and of a deodorant (a body capable of absorbing or fixing foul gases but exercising no control over microbes).

In medical and surgical work the use of disinfectants is acknowledged on all hands. The germ theory of the origin of certain diseases is a necessary article of modern medical faith, but the *modus operandi* of many disinfectants is not always clearly understood, which lack of knowledge leads to frequent and grievous errors. In connection with this matter the truth is that great laxity and carelessness has attached to the production and sale of disinfectants in general, and that, consequently, numbers of unsuitable and worthless bodies have taken possession of the market. An ideal disinfectant is not necessarily a body, as some would seem to think, smelling strongly of coal-tar and capable of removing the epidermis from the hands. An ideal disinfectant for surgical work might rather be defined as a non-toxic, easily-soluble body, which possesses definite germicidal properties, but is incapable of coagulating the protoplasm of the tissues with which it is brought in contact. In order to carry out disinfection intelligently it is clear that cognisance must be taken of differences in the physical and chemical conditions surrounding objects to be disinfected, and of differences in the physical and chemical properties of the agent used. No disinfectant approaches the ideal that exerts a deleterious influence on the tissues, clothing, and what not, to which it is applied. Further, for the intelligent application of disinfection it is necessary to possess practical knowledge of the life histories and characters of the various germs to be destroyed. It is now well known that some germs are easily killed, whilst others are much more resistant; the germicide, therefore, indicated in the first instance, may be of no value whatever in the second. Since safety can only be guaranteed in the entire absence of disease-producing microbes, and when it is remembered how rapidly many germs reproduce themselves, it is obvious that the necessity for early application of a suitable disinfectant is imperative.

Substances like perchloride of mercury and carbolic acid which rapidly coagulate albumen are far from ideals in the disinfection of sputum containing masses of lung tissue in which are embedded numbers of tubercle bacilli. The application of these bodies to such portions of highly infected tissue fails utterly. Their coagulative properties prevent them penetrating the interior of solid pellets, and virulent bacilli are merely enclosed in a shell of coagulated protoplasm, and thus preserved indefinitely from destruction. Yet we frequently find both substances strongly recommended for this work. An elementary knowledge of the chemical properties of proteids would forbid the use of all such and at the same time suggest the proper type of agent. An alkaline emulsion, such as a solution of a soap, will dissolve the albuminoid pellet, instead of coagulating its superficial layers and lay bare the enclosed bacilli.

Such indiscriminate and careless use of disinfectants is an abuse of them.

In the light of present knowledge it is necessary therefore that everyone engaged in all forms of medical and surgical disinfection should possess intelligent views upon the nature of the particular work to be done, and of the type of agent required to effect it. This statement cannot be too thoroughly brought home to the minds of hospital and municipal authorities.

It is encouraging to note that recently a movement has taken place in the direction of procuring a standardisation of disinfectants upon strictly scientific lines which must eventually bring about a complete separation of the wheat from the chaff. By direct appeal to experiments on living bacteria the real germicidal powers of a compound can alone be ascertained. The chemical purity of a given preparation is no guarantee of its bactericidal value. Comparative merits amongst disinfectants can only be obtained by subjecting them individually to the same bacteriological tests, under exactly similar conditions in every respect.

In the Rideal-Walker method of testing disinfectants we possess an unfailing criterion by which may be accurately estimated the germicidal capacity of any body, by putting it directly in competition with a standard whose germ-destroying powers are known. These observers have selected pure carbolic acid as their standard, and, in performing their test, apply measured quantities of the two competing substances to the same quantity of a particular culture of a selected micro-organism, for and at the same time, and at the same temperature. Equal volumes of the medicated culture are then sown in equal quantities of the same medium, at the same temperature, and incubated, for the same time, at the same incubation temperature. Throughout the entire series of steps of the examination the conditions—physical, chemical, and biological—are in the two cases strictly identical, and therefore strictly comparable. The absence or presence of life in the last culture-medium at the end of a certain time, for the most part 48 hours, is the index of efficiency, or the lack thereof. By this method a particular dilution of a disinfectant under test can easily be found that performs the same germicidal work as a particular dilution of the standard. These dilutions are then written in the form of a fraction with the standard as denominator; the ratio obtained is called the carbolic acid coefficient.

The method is the best that has yet appeared, and is now being adopted.

With such means of determining the germicidal values of disinfectants confidence will increase in the utility of disinfection, and as the older casual methods disappear and are replaced by such scientific procedure the gain to Preventive Medicine will be correspondingly great.

